# A Non-Intrusive Approach for Indoor Occupancy Detection in Smart Environments

**DOI:** 10.3390/s18113953

**Published:** 2018-11-15

**Authors:** Bruno Abade, David Perez Abreu, Marilia Curado

**Affiliations:** Department of Informatics Engineering, University of Coimbra, Polo II-Pinhal de Marrocos, 3030-290 Coimbra, Portugal; bruno.abade@student.uc.pt (B.A.); marilia@dei.uc.pt (M.C.)

**Keywords:** smart environments, Internet of Things, indoor occupancy, machine learning, data analysis

## Abstract

Smart Environments try to adapt their conditions focusing on the detection, localisation, and identification of people to improve their comfort. It is common to use different sensors, actuators, and analytic techniques in this kind of environments to process data from the surroundings and actuate accordingly. In this research, a solution to improve the user’s experience in Smart Environments based on information obtained from indoor areas, following a non-intrusive approach, is proposed. We used Machine Learning techniques to determine occupants and estimate the number of persons in a specific indoor space. The solution proposed was tested in a real scenario using a prototype system, integrated by nodes and sensors, specifically designed and developed to gather the environmental data of interest. The results obtained demonstrate that with the developed system it is possible to obtain, process, and store environmental information. Additionally, the analysis performed over the gathered data using Machine Learning and pattern recognition mechanisms shows that it is possible to determine the occupancy of indoor environments.

## 1. Introduction

The Internet of Things (IoT) paradigm enables the interaction between physical objects via application services to add characteristics such as network connectivity, sensing, and actuation allowing to move forward to the Smart Objects approach. Thus, Smart Objects can communicate with each other, share information, and coordinate their actions in order to take smart and cognitive decisions according to the environment where they are deployed [1].

Combining the IoT paradigm and the Smart Objects approach, the concept of Smart City arose. A Smart City uses a variety of sensors and Smart Objects embedded on traditional things and locations (e.g., buildings, parks, and sidewalks) to improve the citizens’ quality of life. One of the Smart Cities sectors is Smart Environments, and its definition is given by Cook et al. “A Smart Environment can acquire and apply knowledge about the surroundings and its inhabitants to improve their experience in that ambiance” [2].

Smart Environments have become popular in recent years targeting the automation of everyday tasks in order to improve the quality of life. A typical example of this kind of systems is the management of energy consumption and Heat Ventilation and Air Conditioning (HVAC) [3,4] in Smart Buildings. For the previous particular use case, it is essential to know the occupancy estimation of specific areas in order to trigger the proper actions to minimise consumption during periods of vacancy, optimise ventilation dynamically for occupant comfort, or forecast of long-term behaviors.

To empower the Smart Environment approach, the use of learning mechanisms plays a key role to analyse patterns, predict situations, and take decisions/actions. Thus, new terms such as Ambient Intelligence (AmI) arise in this context. AmI brings intelligence to our everyday environments, making them sensitive to us. AmI’s primary goal is to introduce automation into the environment to generate knowledge about the users and their surroundings, accumulating data and taking smart and cognitive decisions [5]. Making these environments smarter, we can make the life of their occupants simpler and more automated.

A specific research topic framed in the context of a Smart Environment is focused on looking at people, detecting, tracking and identifying them, as a way to offer high-quality, intelligent services, while considering human factors such as life patterns, health, and mood of a person [2]. One example is to analyse patterns of an elderly person and generate an alert when something abnormal happens. For this, knowing the place’s occupancy is a priority. Many techniques are developed to detect the presence of people, the most common are cameras and wearable devices. However, these devices suffer from privacy or intrusiveness issues. Research challenges arise with the design of occupancy detection techniques. One of these challenges is related to how to preserve occupants privacy. A Smart Environment system should be designed to avoid identifying occupants or their activities. Thus, there is the need for non-intrusive techniques to detect occupancy or improve the mechanisms already available.

Environmental data are an excellent source of information for occupancy detection since the presence of living beings affects the surroundings through heat or Carbon Dioxide (CO${}_{2}$) emission without jeopardising the privacy of the occupants in that particular location. Nevertheless, only with data, it is almost impossible to gauge something. Machine Learning (ML) techniques look at the data and try to find patterns; with these patterns, it is possible to affirm the occupancy with a certain percentage of certainty. Although some contributions have been performed in this direction, there is still room for improvement, and this research proposal is focused on that.

The main purpose of this research is the design and development of an affordable and non-intrusive solution to improve occupants experience in Smart Environments with ML support. The proposed solution monitors temperature, light intensity, noise, and CO${}_{2}$ to estimate the presence of occupants through these environmental features that can be integrated with other existent approaches. First, the data are collected and analysed, before applying ML techniques to infer the occupancy of the area under monitoring. In the first stage, our solution detects the presence or absence of occupants. In the second stage, the number of occupants inside the area of interest is estimated.

This paper is structured as follows. Section 2 discusses the related work. Section 3 presents the solution developed to detect occupants, its architecture, and the key features that were considered, the gathering system and the ML concerns. Section 4 shows the experimental implementation. Section 5 analyses and discusses the results obtained. Finally, conclusions are presented in Section 6 as well as suggestions for future works.

## 2. Related Work

**Figure 1:**
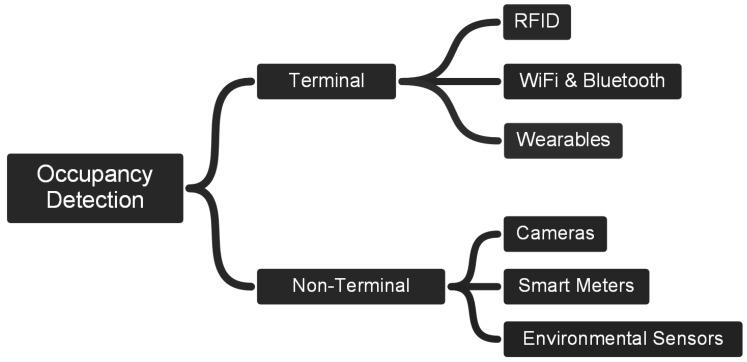
A simple classification of occupancy/location detection methods.

Occupancy detection systems could be classified according to the need to use a terminal or not [6,7]. In the case of the methods that require a terminal, it is necessary to attach a device to the occupants to keep track of them (e.g., a smartphone). In the non-terminal methods, the detection is based on a passive approach that is focused on monitoring areas or spaces instead of the identification of devices (e.g., cameras monitoring a room). Figure 1 depicts a simple classification of the occupancy detection methods following the terminal and non-terminal approaches, and their more specific characterisations, which are used to organise the discussion of this section.

In the branch of terminals, the methods for occupancy and counting lay on devices that have embedded wireless transmitters which support different communication technologies, such as, Radio-Frequency Identification (RFID), WiFi and Bluetooth, or Global Positioning System (GPS) in the case of wearable devices. On the other hand, the branch of non-terminals relies on monitoring specific surroundings by using cameras, smart meters for energy consumption, or environmental sensors (e.g., CO${}_{2}$ and temperature). A discussion of some relevant works on occupancy detection is presented below.

Hahnel et al. [8] proposed a probabilistic measurement model for RFID readers that allows accurately tracking RFID tags in the environment; specifically, the authors studied the problem of localising the RFID tags using a mobile platform based in robots equipped with RFID antennas. Li and Becerik-Gerber [9] performed a survey of RFID-based solutions and the algorithms used for occupancy and location at indoor environments. After discussing more than twenty projects, authors identify the drawbacks of each solution to move forward to the identification of the most relevant research challenges regarding outdoor/indoor location sensing solutions. In a follow-up research, Li et al. [10] proposed an energy-saving strategy for Smart Buildings based on RFID occupancy detection to support demand-driven HVAC operation by detecting and tracking occupants around areas of interest inside the buildings. The use of RFID technologies for occupancy detection is an affordable option considering the price of receptors and tags; nonetheless, this approach could be affected by electric and magnetic conditions of the environment leading to inaccurate occupancy detection. A more constraining issue is the fact that occupants have to carry a special tag to be monitored, making the process invasive and susceptible to additional errors in case the occupants forget their specific devices somewhere.

Some occupation detection methods take advantage of the communication technologies embedded in devices commonly used by the occupants of the area of interest, such as Smartphones, Smartwatches, and Fitness trackers. Huh and Seo [11] came up with a system that estimates the indoor position of a user taking advantage of some specific characteristics of the Bluetooth protocol. Specifically, the system uses beacon frames to extract information about the Received Signal Strength Indication (RSSI) and trilateration that is processed to infer indoor positioning. Filippoupolitis et al. [12] evaluated how accurate occupancy estimation in indoor environments using Bluetooth Low Energy (BLE) could be in a prototype system composed of BLE beacons, a mobile application, and a server. After performing the analysis of the data collected using three ML approaches (i.e., k-nearest neighbors, Logistic Regression, and Support Vector Machine), the authors concluded that the combination of BLE and ML leads to a good occupancy estimation.

Depatla et al. [13] proposed a framework for counting the total number of people walking in an area based on the WiFi RSSI measurements between a pair of transmitter/receiver antennas. The authors developed a mathematical model to determine the probability distribution of the received signal amplitude as a function of the total number of occupants based on Kullback–Leiber divergence estimation. The results obtained concluded the authors’ approach could estimate the total number of people in indoor and outdoor areas with good accuracy. Balaji et al. [14] designed a system, Sentinel, that leverages in the WiFi infrastructure deployed in the area of interest along with Smartphone carried by occupants to estimate occupancy and enhance the performance of the HVAC system via actuation. The Sentinel system proposed by the authors’ shows an accuracy of 86%, with 6.2% false negative error regarding the occupancy in indoor environments. Additionally, the tests performed depict that using actuation over the HVAC system it was possible to save around 17.8% energy.

Wearable electronics, such as Smartwatches and Fitness trackers are becoming more ubiquitous and carrying more sensors and communication interfaces. Jin et al. [3] took advantage of the previous statement to investigate the causal influence of user activity on various environmental parameters monitored by occupant-carried multi-purpose sensors. Their results showed that the fusion of the data collected from the sensors available in the wearable devices (e.g., light level, accelerometer, heart rate, Bluetooth, and GPS) achieves a good classification regarding occupancy/location reaching in some cases values around 99% of accuracy. The quality of data obtained using the method that involved wearable devices, WiFi and Bluetooth allows a more accurate occupancy/location estimation; however, these approaches have privacy concerns regarding how to use the data gathered. For example, the use of Bluetooth allows having access to specific and unique information of the devices, such as the MAC address; or in the case of a Fitness tracker the heart rate histogram could reveal some particular condition or disease. This information could be crossed with other data to obtain detailed information about the owner of the device.

In the non-terminal branch for occupancy/location, the methods that use cameras are well-known. Fleuret et al. [15] combined a generative model with dynamic programming to track occlusions and lighting changes in frame images in order to derive the trajectories of each of them. With the proposed model, authors were able to track multiple persons and ranked their trajectories inside the area under study. Alahi et al. [16] addressed the problem of localising people in crowds using a network of heterogeneous cameras by formulating a problem focused on calculating the occupancy vector per each captured frame; this is the discretised occupancy of people on the ground from the foreground silhouettes. The occupancy approach proposed is complemented by a graph-driven tracking procedure suited to deal with the temporal dynamics of people occupancy vectors. The main outcome of this work is a well-defined mathematical formulation to locate people via cameras that record frames with very noisy features. In the same way as with the wearable solutions discussed above, the use of cameras for occupancy/location brings a set of privacy issues regarding the identity of occupants and objects that could represent a problem in the final solution.

A different solution based on Smart Meters is presented by Chen et al. [17]. They tried to predict the occupancy analysing electrical usage. They observed that the home’s pattern of electricity usage changes when there are occupants. The study was carried out in two homes and later on correlated with statistical data (e.g., power’s mean and variance). Some challenges on non-intrusive occupancy monitoring are also discussed. Another solution by Lee et al. [6] used an array of pyroelectric infrared sensors (PIR) to detect resident’s location in a Smart Home. The authors also proposed an algorithm to analyse the information collected from the PIR sensors. The evaluation was carried out using an experimental testbed. Jin et al. [18] tested several binary techniques using data from residential and commercial buildings based on information regarding power usage that requires minimal system calibration and setup, while also ensuring the privacy of the occupants. The accuracy of these works to determine the number of occupants is low and could be considered just an estimation since the power consumption is aggregated, consequently, the exact number of people occupying the area of interest could not be accurate.

The results of Dong et al. [19] indicate that CO${}_{2}$ and acoustic parameters have the most significant correlation with the number of occupants in a space. Several studies correlate the CO${}_{2}$ concentrations with the presence of occupants such as in the research of Gruber et al. [20]. Ryu et al. [21] used indoor and outdoor CO${}_{2}$ concentrations and electricity consumptions of lighting systems in a controlled testbed. Although the CO${}_{2}$ levels could be used to determine occupancy, gathering this kind of data with good levels of accuracy is not an easy task considering that aspects such as room ventilation, room flow-rate, and presence of plants in the room could drastically influence the concentration and dissipation of the aforementioned gas. Thus, it is not feasible to only use CO${}_{2}$ as a metric for occupancy estimation.

Candanedo et al. [22] and Amayri [23] estimated occupancy using a combination of heterogeneous sensors. The first research uses data from light, temperature, humidity and CO${}_{2}$ sensors; and the second one uses data from luminance, temperature, humidity, motion detection, power consumption, and CO${}_{2}$, as well as data collected from a microphone or door/window burglary sensors. These works follow the same ground truth strategy focused on cameras to corroborate the presence of occupants, which introduce several privacy constraints. Additionally, both works utilise ML techniques to evaluate the results of the proposed solutions, particularly, decision tree learning algorithms. Considering the number of sensors and devices used in these studies were significantly high, our proposal is focused on answering the question whether it is possible to obtain similar or better results regarding occupancy detection using fewer resources. Even more, our proposal uses a non-intrusive ground truth strategy to avoid jeopardising the privacy and security of the occupants in the area of interest.

This research uses as inspiration some of the ideas proposed in the works discussed, in particular, the gathering of data from different sources to move forward to a complete analysis of the data collected using a ML approach. Our goal is to detect occupancy in indoor environments by pre-processing the datasets collected before applying ML to a binary and multi-class problem. Additionally, we design our solution focused on two main requirements: the first one is to try to take advantage of cheap/affordable devices commonly deployed in Smart Environment, and the second one is to guarantee that the privacy of the occupants in the area under study would not be compromised. Thus, after the discussion of the works in this research area, our proposal uses environmental features via the combination of data gathered from different sensors. This solution is presented in the following section.

## 3. Occupancy Detection in Indoor Environments

As discussed in the previous section, occupancy detection could be used to trigger some actuation mechanisms in Smart Environments in order to improve resource usage and user experience, among other factors. An important issue that must be considered is the preservation of privacy of the data collected and analysed. Additionally, it would be desirable to take advantage of the infrastructure available in the surroundings to avoid incurring in extra expenses, while allowing the scalability of the solution. Considering these factors, this research is focused on a non-intrusive and inexpensive solution for occupancy estimation that ensures occupants privacy while taking advantage of the technological infrastructure already available in common Smart Environments including Smart Buildings and Smart Homes. From the analysis performed on Section 2, and to comply with the previously established requirements, our occupancy detection solution is focused on environmental data.

A scene analysis approach is used in this research to extract the features of interest for indoor scenarios to proceed and then to estimate the occupancy in the area using the gathered data [24]. The scene analysis method does not rely on any theoretical model or specific hardware; however, it requires a preliminary phase for capturing features which are influenced by changes in the area of interest [25].

In this section, we explain the criteria applied to select the features used in our solution before moving forward to the description of the design of the four-layers architecture adopted for the gathering and processing of the data. The section concludes with the discussion of the ML classifiers that were selected to improve the performance of occupancy detection in indoor environments. Table 1 summarises the terms used in the remaining of the manuscript.

### 3.1. How Do Human Beings Change Their Surrounding?

A human body is similar to a machine, as to perform actions it needs energy. The first and second laws of thermodynamics state that it is impossible to create energy out of nowhere and a hot body transfers its energy to a cold one. Consequently, the human body is subject to these laws. This energy is interchanged with the environment, in the form of heat, can be by sensible heat (conduction, convection, and radiation) or latent heat transfers (evaporation and condensation) [26]. A healthy adult human releases approximately between 100 Watts (in a resting state) and 1000 Watts (in an effort state), equivalent to the heat dissipated by a few laptops [27]. Thus, the heat of an environment is influenced by the number of persons in there.

Similar to heat, CO${}_{2}$ is a side effect of the metabolism. It is an essential gas for the existence of life, but at very high concentrations (e.g., greater than 5000 parts per million (ppm)) it can pose a health risk. CO${}_{2}$ concentrations commonly found in buildings are not a direct health risk, but this concentration can be used as an indicator of occupancy [28]. In fact, occupants are the principal source of CO${}_{2}$ increasing in indoor environments [29].

In 1879, Thomas Edison invented the first light bulb which made viable to extend the working hours of the human beings [30]. Nowadays, it is common to have artificial light in working spaces. This fact enables the possibility of drawing a relation between light sources and occupancy in indoor environments.

Noise is another feature that is affected by the number of human beings in an ambient [31]. Thus, it is possible to expect that the noise of a specific place will increase with the number of people there. An important fact that should be considered in indoor environments is that they usually have background noise produced by household appliances or other static sources of sound.

Considering the discussion and facts addressed so far in this section, this research uses the following environmental features to detect and estimate the occupancy of indoor environments: heat (via the measurement of the temperature in the area of interest), CO${}_{2}$, light intensity, and noise. Specifically, a testbed was designed to acquire the data and extract the information to be analysed using a ML approach.

In the next subsection, the architecture designed and used to process the data gathered from the features selected in this work is presented.

### 3.2. Data Processing Architecture

**Figure 2:**
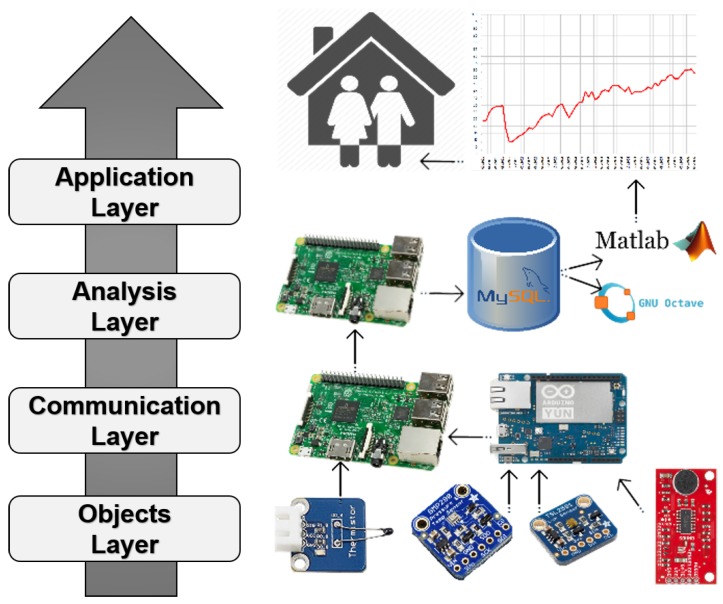
Data processing architecture.

The data processing architecture used in this research is depicted in Figure 2. The architecture has four layers: Objects Layer, Communication Layer, Analysis Layer, and Application Layer. The functionality of each layer is presented below:

- *Objects Layer*: Deals with the physical sensors that collect raw data information. The sensors used in this research are presented in Table 2.
- *Communication Layer*: Handles the data coming from the sensors. In this layer, the following components are used: an embedded operating system, signal processors, microcontrollers, and gateway nodes. In this layer, the communication between an Arduino Yun (a microcontroller board based on the ATmega32u4 and the Atheros AR9331) and the sensors (e.g., thermistors and sound sensors) is carried out using 10 bits ADC via an I2C bus. The Arduino Yun communicates with a Raspberry Pi (RPi) by Serial Communication performed by the Universal Serial Bus (USB) port to process and store the data gathered.
- *Analysis Layer*: Provides the data management required to extract the necessary information from the raw data collected in the lower layers. This layer includes the elements to perform data mining, analytics services, and device management. The data collected and analysed are stored using a MySQL Database (version 5.5). The tool used to perform the analytics of the data was Matlab (version v.9.2.–R2017a).
- *Application Layer*: Deals with the utilisation of the processed data. It includes services and applications. This last layer uses the previous layers to acquire raw data through sensors, storing and treating it to apply ML techniques to perform the main goal of this research, which is to detect people in indoor environments using a non-intrusive approach.

The flow of data in the processing architecture begins at the lower level with the raw data acquisition via environmental sensors. Specifically, every time that the Arduino Yun receives a signal from the RPi (every 10 s), the former one gets ten samples with a delay of 100 microseconds. Next, an average is computed and sent to the RPi. Finally, a new average is calculated using the aggregated data of six values and it is stored in the MySQL database. With this average, it is possible to decrease the fluctuations in the data. This process is repeated every minute. In the upper layers of the architecture, the analytic functions using ML are run over the data collected and stored after pre-processing it. Thus, the outcome of the Application Layer will be an estimation of the occupancy of the indoor area under study.

### 3.3. Machine Learning Classifiers and Their Parameters’ Tweaking

In the ML context, a supervised approach is used to process the data so the system could learn from it. We use three classifiers: Logistic Regression (LR), a direct probabilistic interpretation; Support Vector Machine (SVM), a hyperplane with the maximal margin to separate the data with similarities; and Artificial Neural Network (ANN), a classifier inspired by how the human brain works. For the ANN case, the hypothesis function is obtained by processing the input features via a set of activation units. These classifiers are largely used in classification problems [32,33,34]. The purpose of using these three classifiers is to compare and select the best classifier, considering the specific problem and the features involved.

Some parameters can improve the overall performance of the classifier. These parameters have fix/default value results under the same conditions. Thus, it is necessary to tweak the classifiers’ parameters according the problem under study to improve the results obtained during the analysis of the data. For LR, the regularisation parameter λ and the degree of the polynomial pd were used. In the case of SVM, we applied the penalty cost *C*, and the standard deviation parameter γ. For ANN, the number of hidden layers units hu, and the previous λ and pd parameters were utilised.

Regarding the default cases, for LR, a sigmoid function with threshold equal to 0.5, λ=0, and pd=1 was used. For SVM, a Radial-Basis Function (RBF) kernel was used in conjunction with these values C=1 and γ=0. For ANN, three layers (input layer, hidden layer, and output layer) were used, and the following values were set: λ=0, hu=1, and pd=1. These values represent the base cases for each classifier before proceeding with a grid search during the training phase in order to tweak them to improve the performance of each classifier. For LR, the possible values of λ were [0,0.01,0.1,1,10,100] and for pd the range was 1–3. For SVM, γ values were [0,0.01,0.1,1,10], and *C* could assume [0.1,1,10]. Finally, for ANN, λ could take the following values [0,0.1,1,10], the range of values of pd was 1–3, and hu could assume 1–3. To select the default and improved values for the classifiers, we used the recommendations of Clarke et al. [32] and Perez et al. [33].

In this research, two problems were studied, a binary problem, where the positive case is the presence of occupants and the negative case is the absence of occupants, and a multi-class problem, where the objective is to determine the exact number of occupants. The research started with the selection of the features to estimate the occupancy in indoor environments, to move forward to the design of the four-layers data processing architecture corresponding to: (1) the sensors to gather the data concerning the features selected; (2) the communication protocols used between sensors, microcontrollers, and processors; (3) how to pre-process and store the data; and (4) the analysis of the data collected using a ML approach where the different classifiers were tested in order to determine the best one by tweaking their parameters.

The setup of the testbed used in this work, as well as how the ML classifiers were evaluated according to their performance is detailed in the next section.

## 4. Experimental Setting

The experiments were conducted in a room of the Laboratory of Communications and Telematics–Centre for Informatics and Systems located in the middle of the Department of Informatics Engineering at the University of Coimbra. The room has an area of 8.5 × 5.5 m${}^{2}$ and 4.15 m of height. This room has a small occupancy change (the maximum number of occupants is five, and the minimum number of occupants is zero) and very low ventilation. The only ventilation in the room is the door and few window cracks. The heating, ventilation, and air conditioning equipment were off during the time of the tests to prevent any influence on the data collected, and it was assumed that the occupants kept the doors and windows closed and the lights on during the period they were in there.

This testbed was set up to study occupancy detection in indoor environments using non-intrusive sensors and ML techniques. The primary objective of the experiments is to evaluate the accuracy regarding occupancy detection in two branches, the simplest one focuses on the presence or absence of occupants; and the more advanced one on the estimation of the number of occupants. In the remainder of this section, we discuss about the placement of the nodes to collect the environmental data and analysis performed to evaluate the accuracy and precision of the classifier utilised. The datasets used in this research, as well as the source code used in the nodes (e.g., Arduinos and RPIs) and the analysis of the data using ML methods, are available via a GitHub repository [35].

### 4.1. Nodes Placement and Ground Truth Strategy

**Figure 3:**
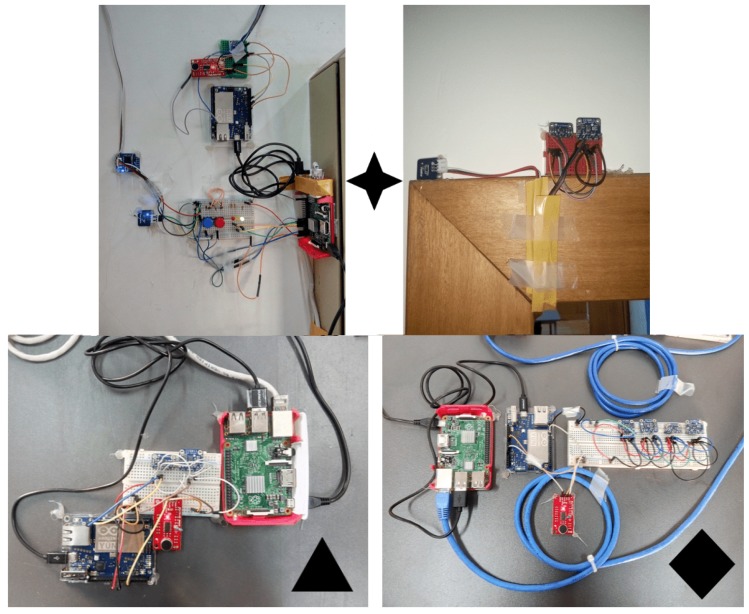
Nodes and sensors deployed in the testbed: (**top**) Node 1 ((**left**) indoor sensors; and (**right**) outdoor temperature sensor); and (**bottom**) Node 2 ((**left**) and Node 3 (**right**).

**Figure 4:**
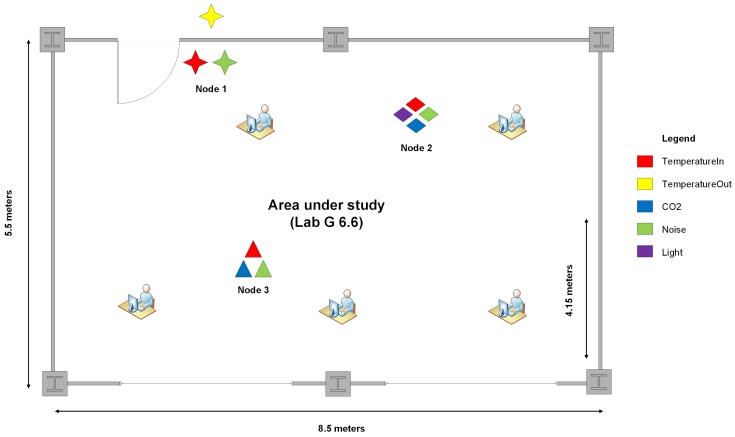
Nodes and sensors placement.

Three gathering and processing nodes (i.e., 3 RPis and 3 Arduinos) were placed in the room (see Figure 3) in strategic positions to collect data. Figure 4 shows the physical location of the nodes in the area under study. Node 1 has a temperature (in and out) and sound sensors; Node 2 has temperature, CO${}_{2}$ and sound sensors; and Node 3 has the most significant variety of sensors including temperature, CO${}_{2}$, sound, and light intensity. Besides gathering environmental data, Node 1 is responsible for controlling the number of occupants in the room (i.e., the ground truth device is attached to it) and also works as the storage node of the data collected during the experiment.

Three CO${}_{2}$ and temperature sensors were placed on each node, and the average of the values collected were computed to mitigate possible fluctuations. The sound detectors were placed close to the occupants for accuracy purposes. The light sensor was placed as far from the windows as possible so that the main light source incident on it was one of the lamps. Regarding the temperature, a sensor was placed in the hallway and other sensors inside the room. The difference between the temperatures gathered at these two different places was analysed to estimate the occupancy in the room.

A ground of truth approach was adopted in this research to validate the data gathered. Concretely, in this work, a simple mechanism with two buttons (blue to enter and red to leave) was developed to create the ground truth and register when a person enters or leaves the room. Every time that an occupant presses one of these buttons, the counter is increased or decreased, respectively. To visualise if the number of occupants is correct, three LED were introduced as a binary counter ($2^{n}-1$ occupants in the room). The leftmost LED is the most significant and the rightmost LED is the least significant. The number of total occupants by minute is the average of samples acquired every 10 s.

### 4.2. Classifier Performance Evaluation

To analyse the performance of the classifiers, several criteria were used. Accuracy measures the percentage of entries that were correctly classified (see Equation (1)), and the miss rate measures the percentage of entries that were incorrectly classified (see Equation (2)) [36]. True Positive (TP) and True Negative (TN) represent the correct classification/prediction if the entry belongs to the positive class or negative class, respectively. False Negative (FN) and False Positive (FP) represent the incorrect classification/prediction if the entry does not belong to the negative and positive classes, respectively [36].

(1) $$Accuracy=\frac{TP+TN}{N}*100$$

where *N* is the total size of the training dataset.

(2) Missrate=(100−Accuracy)

To evaluate a classifier, it is necessary to verify its accuracy when it has to process new data. The classifiers can have a high accuracy when they are tested with the training dataset, but they can have a low accuracy with a new dataset. Thus, it is recommended to split the data into a training dataset and a testing dataset [37]. The training dataset is suitable to train the classifiers, and the testing dataset is appropriated to measure their performance to new entries. Typically, the dataset is divided into three portions: training (to train the classifiers), cross-validation (to adjust the parameters), and testing (to verify the performance of the classifiers) [33,37]. In this work, the dataset used was split following the same approach, particularly for the cross validation the k-fold method with k=5 was used [38].

In certain cases, the dataset can have skewed classes, i.e., one class has a small set of data. For example, assuming that the training dataset contains 0 positive and 100 negative entries, and if all instances are predicted correctly, the accuracy will be 100%, but the classifier had no chance of learning the hidden patterns. With the previous example, it can be said that the accuracy does not work well when the dataset is unbalanced, i.e., it has more data in one class than in the other.

The F-Score was used to predict the performance of the classifiers. It is a technique that measures the discrimination of classes, through a harmonic mean of two metrics, recall and precision (see Equation (5)) [37]. Recall measures the percentage of entries that belongs to the positive class and was classified/predicted correctly (see Equation (3)) [36]. Precision measures the percentage of hits over the entries of the predicted positive class that really belongs to positive class (see Equation (4)) [36]. To have a high F-Score, both precision and recall must be high.

(3) $$Recall=\frac{TP}{TP+FN}*100$$

(4) $$Precision=\frac{TP}{TP+FP}*100$$

(5) $$FScore=2*\frac{Precision*Recall}{Precision+Recall}$$

Equation (5) can only be applied to binary classification problems, but it can be extrapolated to a multi-class classification problem. The Macro-average method takes the average of precision and recall of each class label (see Equations (6) and (7)) [39,40].

(6) $$Recall=\frac{Recall_{1}+Recall_{2}+\ldots +Recall_{k}}{k}*100$$

where *k* is the class label.

(7) $$Precision=\frac{Precision_{1}+Precision_{2}+\ldots +Precision_{k}}{k}*100$$

The analysis and discussion of the results obtained in this research are presented in the next section.

## 5. Results and Discussion

**Figure 5:**
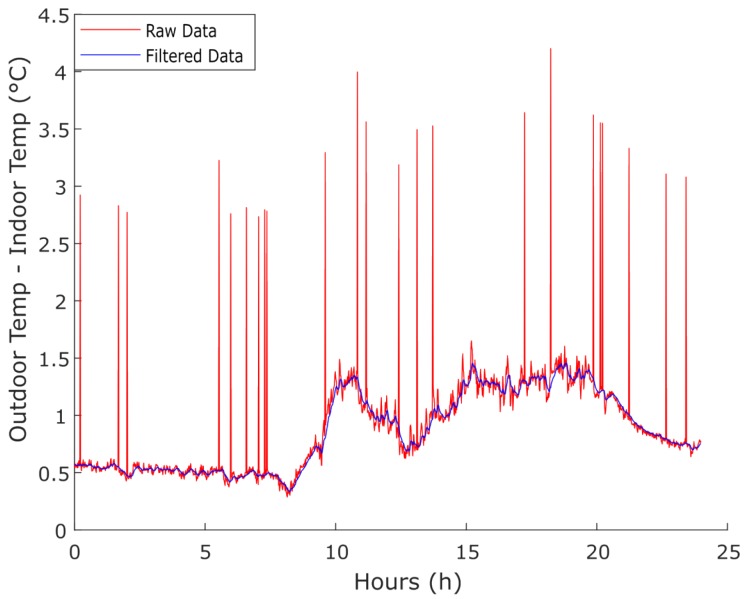
Performance of the outlier and LPF filters over the temperature (i.e., difference of outdoor and indoor temperatures) data gathered.

The data acquisition for this research was performed over two weeks on November 2017 using a rate of one sample per minute. First, the data were analysed and a strategy to use it was defined. It was confirmed that some data had outliers and noise; consequently, to mitigate this issue, two filters (i.e., an outlier filter and a Low-Pass Filter (LPF)) were applied. The performance of the filters over the data is depicted in Figure 5.

An LPF is a circuit that offers easy passage to low-frequency signals and difficult passage to high-frequency signals. Equation (8) gives the discrete implementation of the first order LPF, where α is the smoothing factor, *y* is the filtered output, *x* is the input, and *n* is the sample index. Calculating the next value through this smoothing factor and the previous value, it was possible to reduce the data noise, making the transitions between samples slower and smoother.

(8) y[n]=αx[n]+(1−α)y[n−1]

In a second stage, ten consecutive days of data were selected, representing a total of 14,400 samples (where almost 25% represented positive cases, and 75% represented negative cases) for each dataset and the ML mechanisms were applied. The dataset was divided into two portions, training and testing. The training portion was then subdivided into two portions, training and cross-validation portions, respectively, representing 70% of the original dataset and the remaining 30% corresponds to a testing portion. Within the first portion (i.e., training) 80% was used for training and 20% was used for cross-validation according to the k-fold (with a k=5) approach to prevent overfitting [38]. This value for k=5 was selected given the unbalanced nature of our dataset, where the average occupancy was around 8 h per day that corresponds to office hours; thus, having periods of 16 h without relevant data per day. Using this value (i.e., k=5), we minimise the probability of having k-portions without any relevant data.

In the remaining of the section, a discussion about how the data were pre-processed and the approach used during the binary and multi-class problems is presented.

### 5.1. Data Pre-Processing

**Figure 6:**
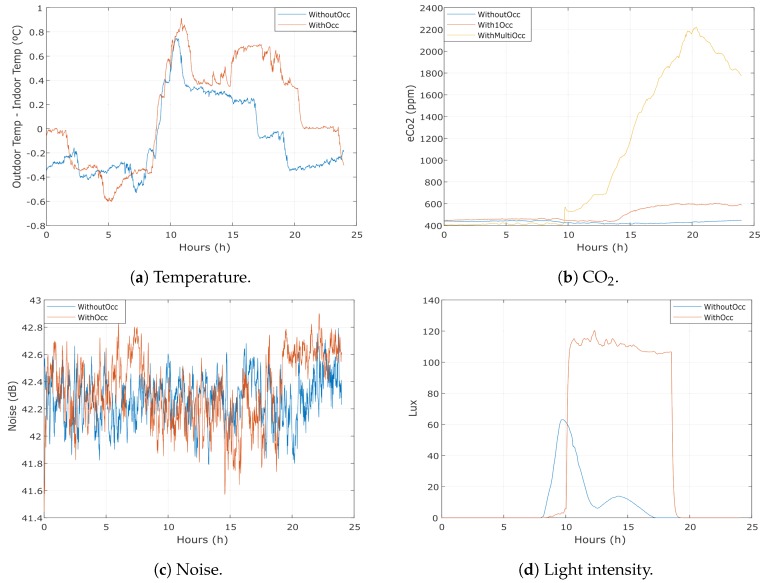
Processing and analysis of the environmental data gathered.

The processing and analysis of the environmental data gathered during the research are depicted in Figure 6a–d. In the charts, the blue and red lines represent a day without and with occupants in the area under study, respectively. Particularly, in Figure 6b, the red line depicts a day with precisely one occupant in the room and the yellow line a day with more than one occupant.

In Figure 6a, the graph is in Celsius degrees by hours. For this analysis, it is important to point out that the data corresponds to the subtraction of the indoor and outdoor temperature as it was described in Section 4.1. For the data collected, it is possible to conclude that the temperature’s difference is higher with occupants than without occupants. The first occupant arrived around 09:00 and the last occupant left around 18:00. There are a couple of exceptions around 10:00 and around 12:00. The first one happens because of the incidence of the sunlight in the room, which on this period of test occurs at this hour, increasing the indoor temperature. The second one occurs when the occupants left the room to have lunch.

In Figure 6b, the graph is in ppm per hour. It is possible to see that when an occupant arrived (i.e., around 09:00) the CO${}_{2}$ levels increased approximately 500 ppm. This increase was more noticeable when more than one occupant was in the room, increasing to around 2000 ppm. In days without occupants, the levels were between 400 and 450 ppm.

Figure 6c depicts the noise data processed. It is possible to see that the differences are not significant considering that the values are almost the same with or without people.

The light intensity data is depicted in Figure 6d. It is possible to see that when an occupant arrived, close to 10:00, the lux increased to around 110 and when he left, close to 18:00, the lux decreased to zero. Around 10:00, it is possible to notice an increase in the light intensity as a result of the incidence of the sunlight in the room. This behavior is consistent with the results and the observations performed during the analysis of the temperature in the room.

After pre-processing the data, each classifier was tested. The results obtained are presented in the next two subsections.

### 5.2. Binary Problem

The binary problem aims to determine whether an occupant was in the room (y=1) or not (y=0). Table 3 presents the results by applying the classifiers with the dataset without changing the parameters. Analysing the results, the classifiers with best F-Scores were LR followed by SVM. The ANN classifier had the lowest F-Score in almost all the cases. In some cases, the result was 0%, i.e., the classifier could not predict any positive outcome.

Regarding the noise as an element to estimate occupancy, the F-Score results show that for the area under study this feature is not a good indicator; considering that the room is a workplace, people are usually concentrated and spend most of the time in silence. The results of the F-Score related to the light intensity were satisfactory with the limitation that this approach could not be used to estimate the number of occupants in the room, just their presence.

As more occupants in the room usually results in higher CO${}_{2}$ concentrations, these data can detect the number of occupants, as can be seen in Figure 6b. However, because the room does not have a good air flow rate, this concentration reduces slowly and can take hours to stabilise. Consequently, this approach to estimate the number of occupants inside the laboratory did not perform as expected. One possible approach to enhance the results obtained would be to calculate the derivative and then check whether it has a certain slope to determine if an occupant arrived or left the room; nevertheless, this method requires more data, as well as more analysis.

The temperature data suffer from the same problem than the CO${}_{2}$ data. It is difficult to have a fixed number of occupants in the room. Thus, it was important to have a dataset with more data for calculating the time taken for the temperature to stabilise to improve the results. However, even without this knowledge, the results were satisfactory (i.e., we obtained in average an F-Score of 89%) to detect the presence of occupants.

Table 4 presents the parameters for which the highest F-Scores were obtained using the LR, SVM, and ANN classifiers to the dataset collected. When performing a new F-Score and changing the parameters and the polynomial degree, some features show an improvement, such as CO${}_{2}$ with an enhancing on the F-Scores of the classifiers between 15% and 47%. Light, temperature, and noise did not show significant growth. Even though the CO${}_{2}$ levels can be used to infer the number of occupants, the data analysed has to suffer changes before applying a ML technique. The noise had a low F-Score, and the light indicated only the presence or absence of occupants. For these reasons, only the temperature was analysed in a multi-class problem.

The F-Scores reached by the classifiers, particularly in the case of Temp and Light, show that it is possible to obtain high accuracy regarding indoor occupancy using the LR, SVM, and ANN classifiers, which is aligned with some of the results reported in the state of the art. Specifically, in the research work of Candanedo et al. [22], a research framed within the same topic although using a different dataset, the authors obtained an accuracy of 85.33% for Temp and 97.66% for Light using a Linear Discriminant Analysis (LDA) model. These results are comparable with the values obtained by LR and SVM in this research considering the classifiers’ linearity. In the case of Temp, LR and SVM showed better accuracy (i.e., around 4%). On the other hand, for Light, LDA performed better than LR and SVM by around 2%. Instead of an ANN approach, Candanedo et al. [22] decided to determine the performance of Classification and Regression Trees (CART) learning algorithms for this specific problem. The accuracy results obtained using CART were 86.51% and 99.31% for Temp and Light, respectively. Thus, for Temp, the ANN approach had a better accuracy 89.72% (i.e., around 3%), and, in the case of Light, the CART model beat the ANN classifier by around 4% (i.e., 99.31% against 95.32%).

### 5.3. Multi-Class Problem

The multi-class problem aims to estimate the number of occupants in a room. During this work, on average, there were five occupants in the room. After observing the behavior of the data gathered and the binary problem results, it is possible to conclude that temperature is the more interesting feature to be tested in the multi-class approach. Even though it was possible to obtain F-Scores beyond 95% for all the classifiers for the data corresponding to light, its binary nature makes impossible to use it to estimate the number of occupants in the area under study, thus it was discarded.

Table 5 summarises both the parameters and the F-Scores obtained using the default values for said parameters, and after tweaking them. Concerning the analysis of the temperature data using the default parameters for each classifier, the following F-Scores results were obtained: 24.43% for LR, 24.90% for SVM, and 25.15% for ANN. These results are far away from what we had expected, thus an additional tweaking of the parameter was applied in order to improve the F-Scores. In the best scenario, the following F-Scores results were obtained: 29.43% (more 5%) for LR; 29.72% (more 4.82%) for SVM; and 28.70% (more 3.55%) in the case of ANN.

The results obtained for the multi-class problem show that it was not possible to estimate the number of occupants using just the temperature data. When the default parameters were used, all the classifiers reported almost the same F-Scores, i.e., around 24.5%. After changing the parameters, the SVM classifier produced the best results for this dataset, around 29.72%. It was also assumed that the human body surface had a uniform temperature and a consistent heat production, but this is not necessarily true. The human body has a distinct physical shape and also has multiple thermo-physiological properties. Thus, it is difficult to include those factors in a numerical constant in an indoor space.

A valid estimation of the number of occupants in indoor environments using non-intrusive environmental sensors requires a deep study of the correlation of the data gathered. ML techniques have proven to be useful to better understand the interaction and behavior between the sensors, according to the changes induced by the occupants in indoor environments. For the multi-class problem, the correlation between light, temperature, and CO${}_{2}$ looks promising. In a first step, the analysis of the light could determine accurately the presence or absence of occupants, and, as a second step, a study of the correlation between temperature and CO${}_{2}$ could enhance the estimation concerning the number of occupants in a specific area. Thus, these open issues lead to the possibility to perform future research in this field.

## 6. Conclusions

Nowadays, companies and researchers are working on enhancing the quality of life of citizens, using the IoT paradigm to reach the idea of building Smart Environments. In this context, it would be beneficial to have mechanisms to predict or estimate the occupancy of indoor environments to make smart decisions about how to self-adapt to the environmental conditions.

In this research, a solution for occupancy detection with non-intrusive devices using sensors such as temperature, noise, CO${}_{2}$, and light intensity was proposed and tested. A functional system, made up of a device to gather and process environmental data, and to analyse the data patterns over the collected data regarding people occupancy in indoor environments using ML methods, was tested. The analysis performed allows asserting that with features such as noise data in working environments the performance of the recognition system might be degraded. However, with features such as temperature, CO${}_{2}$, and light data, it will be possible to estimate the detection of occupants with an acceptable level of accuracy. Thus, the work done in this research could feed third-party applications focused on indoor occupancy detection to generate smart decisions considering the occupants’ needs.

For future works, it is necessary to study the full correlation of the environmental data used in this research. A starting point could be the analysis of features and their impact on the system. The CO${}_{2}$ data have to endure some processing to find the most meaningful way to use this type of data, since it was found that they represent one of the best features to detect the number of occupants.

## Figures and Tables

**Table 1:** Notation table.

Term	Meaning
Temp	Temperature
LR	Logistic Regression
SVM	Support Vector Machine
ANN	Artificial Neural Network
TP	True Positive
TN	True Negative
FP	False Positive
FN	False Negative
λ	Regularisation Parameter
pd	Polynomial Degree Parameter
*C*	Penalty Cost Parameter
γ	Standard Deviation Parameter
hu	Hidden Layers Units

**Table 2:** Sensors used in the Objects Layer.

Name	Type	Manufacturing	Communication
NTC Thermistor Module	Temperature	Adafruit	ADC
CCS811 Breakout	CO${}_{2}$	Adafruit	I2C
Sound Detector	Noise	SparkFun	ADC
TSL2591 Breakout	Light	Adafruit	I2C

**Table 3:** F-Score results of applying ML algorithms to the data collected for the binary problem with default parameters.

	LR	SVM	ANN
Temp	89.70%	89.66%	89.60%
CO${}_{2}$	6.59%	1.43%	0%
Noise	1%	1.28%	0%
Light	95.60%	95.60%	95.42%

**Table 4:** F-Score results of parameters that perform the highest score for LR, SVM, and ANN for the binary problem.

	LR			SVM			ANN			
	λ	pd	F-Score	γ	C	F-Score	λ	pd	hu	F-Score
Temp	0	2	89.80% (+0.10%)	0.1	1	89.71% (+0.05%)	0.1	1	2	89.72% (+0.12%)
CO${}_{2}$	0	3	22.10% (+15.51%)	10	10	43.98% (+42.55%)	0	1	2	47.81% (+47.81%)
Noise	0	2	2.60% (+1.60%)	10	10	4.17% (+2.89%)	0	1	3	0% (+0.00%)
Light	10	1	95.60% (+0.00%)	1	0.1	95.55% (+0.13%)	1	1	1	95.32% (−0.10%)

**Table 5:** F-Score results of parameters for LR, SVM, and ANN for the multi-class problem.

	LR			SVM			ANN			
	λ	pd	F-Score	γ	C	F-Score	λ	pd	hu	F-Score
Default	0	1	24.43%	0	1	24.90%	0	1	1	25.15%
Tweaked	0.01	2	29.43% (+5%)	10	10	29.72% (+4.82%)	0	1	3	28.70% (+3.55%)
